# Eosinophil count and tumor necrosis factor α in response to phototherapy treatment of neonatal hyperbilirubinemia: a cross sectional study

**DOI:** 10.1186/s13052-022-01302-w

**Published:** 2022-06-20

**Authors:** Mai Rabie El-Sheikh, Amira Youssef Ahmed, Abd EL-Rahman Mohamed ELMashad, Ibrahim Ibrahim Talaye, Eslam El-Sayed El-Hawary

**Affiliations:** 1 Pediatric and Neonatology, Faculty of Medicine, Tanta University, El Bahr St.، Tanta Qism 2, Tanta, 31527 Gharbia Governorate Egypt; 2 Clinical Pathology Department, Faculty of Medicine, Tanta University, Tanta, Gharbia Governorate Egypt; 3 Ministry of Health, Tanta, Egypt; 4 Pediatric Hematology Department, Faculty of Medicine, Tanta University, Tanta, Egypt

**Keywords:** Neonatal hyperbilirubinemia, Phototherapy, Allergic response, Eosinophilic count, Tumor necrosis factor-alpha

## Abstract

**Background:**

Phototherapy (PT) is the most often utilized technique for treating and preventing severe hyperbilirubinemia in the term and preterm newborns. PT's proven benefit is that it decreases the requirement for exchange transfusions. To investigate the effect of PT on allergic response mediators in neonates with hyperbilirubinemia treated by PT, eosinophil counts and tumor necrosis factor alfa levels have been assessed.

**Methods:**

This cross-sectional study included 100 full-term infants with indirect hyperbilirubinemia in the first two weeks of life who were indicated for PT. They were investigated by tumor necrosis factor α and eosinophil counts before and 72 h after starting PT. The used tests were paired with Student’s t-test and Pearson coefficient.

**Results:**

Relative and absolute eosinophil counts and tumor necrosis factor alfa were significantly higher after PT than before (*p* < 0.001). There was a significant positive correlation between total serum bilirubin and both tumor necrosis factor alfa and eosinophil % (*r* = 0.442 and *r* = 0.362, respectively, *P* < 0.001) before PT. There was a significant positive correlation between total serum bilirubin and both eosinophil count and eosinophil % (*r* = 0.281and *r* = 0.339), respectively (*P* < 0.001) after PT. There was a significant positive correlation between both tumor necrosis factor alfa and eosinophil % after PT (*r* = 0.545, *P* < 0.001)**.**

**Conclusions:**

Serum tumor necrosis factor-alpha and eosinophilic count increased after treatment of neonatal hyperbilirubinemia by PT, which indicates an allergic response to PT in neonates.

## Background

Neonatal hyperbilirubinemia is one of the most prevalent conditions seen daily by neonatologists. Around 60% of term newborns and 80% of preterm infants develop jaundice within the first week of life [1]. Neonatal hyperbilirubinemia may arise from physiological or pathological causes [2]. Phototherapy (PT) is the most often utilized technique for treating and preventing severe hyperbilirubinemia in term and preterm newborns. PT's primary proven benefit decreases the requirement for exchange transfusions [3].

As with any treatment, PT may cause adverse effects such as hyperthermia, food intolerance, loose stools, skin rashes, dehydration, hypocalcemia and blood flow redistribution [4]. Additionally, PT may cause-specific long-term adverse effects, including melanocytic nevi, skin cancer, patent ductus arteriosus, and retinal impairment [5]. NNPT degradation of bilirubin may increase oxidative stress, a possible risk factor for asthmatic manifestations on later life [6]. Thus, the decreased bilirubin level induced by NNPT and the resulting impaired antioxidant defense may contribute to the development of asthma.

History of exposure to PT during the neonatal period was listed among the potential risk factors for childhood asthma [7]. It was also found to have a strong association with allergic rhinitis and conjunctivitis [8]*.* This was explained by the PT 's ability to influence the synthesis and release of cytokines from the peripheral immune system as interleukin IL-1, IL-6, IL-10, and tumor necrosis factor-alpha (TNF-alfa) [9]*.*

It was noticed that, at 72 h of exposure to PT serum, TNF-alfa, IL-1beta and IL-8 levels were increased. In addition, the percentage of CD3 + lymphocyte subset is significantly lower in newborns at 72 h of exposure to PT [10]*.*

PT also causes direct DNA damage to lymphocytes in jaundiced infants [8] and the DNA damage increases with the increasing duration of PT. These changes in cytokine levels and DNA damage to lymphocytes may contribute to the imbalance in T helper cells subpopulations (Th-2/Th-1 switch disorder) [11]. Abnormalities in the Th-2/Th-1 switch caused by environmental factors, including PT, can contribute to many allergic diseases [12]***.***

The present study was established to compare eosinophil counts and levels of serum TNF-alfa before and 72 h after starting PT for infants with neonatal hyperbilirubinemia.

## Methods

This cross-sectional study was established on 100 full-term infants presented with indirect hyperbilirubinemia in the first two weeks of life and indicated for PT referred to Neonatal Intensive Care Unit (NICU), Pediatric Department, Tanta University Hospitals, Egypt. The study was done after obtaining approval from the ethical committee of the Faculty of Medicine, Tanta University, Egypt (33,344/19/9). All methods were carried out in accordance with the ethical standards as laid down in the 1964 Declaration of Helsinki and its later amendments or comparable ethical standards. Informed consent from one of the parents and/or legal guardians.

We excluded premature babies and infants with one or more conditions: direct hyperbilirubinemia, birth injuries, congenital malformations, congenital infections, birth asphyxia, and neonatal sepsis.

All studied infants underwent full medical history taking and thorough clinical examination. Routine investigations were done, including liver function tests, renal function tests, reticulocyte count, and RH and ABO blood grouping. We specifically studied the complete blood count parameters, emphasizing neutrophils count and serum levels of TNF-alfa in samples collected before and 72 h after starting PT.

### Sample size

The sample size calculation was performed using G.power 3.1.9.2 (Universitat Kiel, Germany). The sample size was calculated as N ≥ 82 based on the following considerations: 0.05 α error and 95% power of the study to demonstrate eosinophile before PT with a mean value (± SD) (0.54 ± 0.30) and after PT with a mean value of (0.67 ± 0.34) (the primary outcome) [13] according to a previous study). Twelve cases were added to overcome dropout. Therefore, 100 patients were enrolled.

### Statistical analysis

Statistical analysis was done by SPSS v26 (IBM Inc., Chicago, IL, USA). Qualitative variables were presented as frequency and percent (%). Quantitative variables were presented as mean and standard deviation (SD) and compared the two groups utilizing paired Student's t-test. Pearson coefficient was performed to correlate between two normally distributed quantitative variables. *P*-value ≤ 0.05 with two tails was considered statistically significant.

## Results

There were 60% males and 40% females among the studied babies. 80% were delivered by cesarean section, while 20% were by vaginal delivery. Regarding the cause of jaundice, 60% had physiological jaundice, while the remaining cause was ABO incompatibility, RH incompatibility and mixed ABO and RH incompatibility (28%, 8%, and 4%, respectively). In this study, there was a non-significant difference between the main cause of hyperbilirubinemia, physiological jaundice representing the 60% and pathological jaundice the remaining 40% (Table 1).

**Table 1:** Relationship between types of jaundice and delta TSB, eosinophil count, eosinophil % and TNF-alfa

	Physiological (*n* = 60)	ABO (*n* = 24)	RH (*n* = 8)	RH & ABO (*n* = 4)	Breast milk (*n* = 4)	*P* value
Delta TSB	8.02 ± 2.21	6.96 ± 2.06	7.30 ± 1.52	8.0 ± 0.94	6.30 ± 0.84	0. 167
Delta eosinophil count	-129.27 ± 69.34	-143.17 ± 76.52	- 16.12 ± 52.35	-167.75 ± 58.11	-200.0 ± 90.226	0.245
Delta eosinophil %	1.47 ± 0.72	1.67 ± 0.76	1.00 ± 1.06	1.00 ± .000	2.00 ± .000	0.083
Delta TNF	54.13 ± 43.10	66.53 ± 31.06	32.28 ± 6.57	52.71 ± .01	25.96 ± .02	0.124

*TSB* Total serum bilirubin, *TNF α* Tumor necrosis factor α, Delta: comparison between before and after measurements

The mean gestational age was 38.44 ± 1.03 weeks and the mean postnatal age at the time of admission was 3.36 ± 1.6 days.

When we compared the CBC parameters before and after PT, we found a significant decrease in RBCs, Hct %, and Hb (*P*. value < 0.001), while there was an insignificant difference as regards the total leukocyte count (*P*. value = 0.105). Platelet count was significantly decreased after PT than before (*P* value = 0.001) (Table 2).

**Table 2:** CBC before and after PT

	Before (*n* = 100)	After (*n* = 100)	Paired t-test	
			T	*P*-value
RBCs (× 10^6^/ul)	4.85 ± 0.84	4.24 ± 0.71	9.969	< 0.001*
Hct (%)	49.03 ± 7.85	43.52 ± 6.88	10.834	< 0.001*
Hb (g/dl)	17.04 ± 2.50	15.35 ± 2.09	13.689	< 0.001*
WBCs (× 10^3^/ul)	10.94 ± 3.16	11.29 ± 2.89	1.634	0.105
Platelet count (× 10^3^/ul)	302.47 ± 91.47	274.1 ± 72.14	2.641	< 0.001*

*Hb* Hemoglobin, *Hct* Hematocrit test, *RBCs* red blood cell, *WBCS* White blood cell, *: significant as *p*-value ≤ 0.05

The analysis of the relative and absolute eosinophil counts was significantly higher after PT with a mean value (336.96 ± 137.23) than before PT with a mean (173.32 ± 80.01) (*P*. value < 0.001) (Table 3). TNF-α was also significantly higher after PT with a mean value (168.85 ± 163.25) than before PT with a mean value of (56.91 ± 37.05) (*P*. value < 0.001) (Table 3)*.* Total, indirect, and direct serum bilirubin were significantly lower after PT than before PT (*P*. Value < 0.001) (Table 4).

**Table 3:** Serum bilirubin before and after PT

	Before (*n* = 100)	After (*n* = 100)	**Paired t-test**	
			T	*P*-value
**TSB (mg/dl)**	17.21 ± 1.74	8.85 ± 0.62	45.812	< 0.001*
**Indirect serum bilirubin (mg/dl)**	16.36 ± 1.64	8.34 ± 0.68	45.633	< 0.001*
**DSB (mg/dl)**	0.85 ± 0.31	0.51 ± 0.21	11.513	< 0.001*

*TSB* Total serum bilirubin, *DSB* direct serum bilirubin, *: significant as *p*-value ≤ 0.05

**Table 4:** Eosinophil count, % and tumor necrosis factor α (TNF-α) before and after PT

	Before (*n* = 100)	After (*n* = 100)	**Paired t-test**	
			T	*P*-value
Eosinophil count (cells/ul)	173.32 ± 80.01	336.96 ± 137.23	18.801	< 0.001*
Eosinophil %	1.60 ± 0.64	3.12 ± 1.40	14.230	< 0.001*
TNF α (pg/ml)	56.91 ± 37.05	168.85 ± 163.25	7.314	< 0.001*

*TNF α* Tumor necrosis factor α, *: significant as *p*-value ≤ 0.05

There was highly statistically significant positive correlation between total serum bilirubin (TSB) and both TNFα, eosinophil % before PT (*r* = 0.442, *P* value < 0.001), (*r* = 0.3618, *P* value < 0.001) respectively. There was highly significant positive correlation between both TNF-α and eosinophil % after PT (*r* = 0.545, *P* value < 0.001). There was highly significant positive correlation between TSB and both eosinophil count, eosinophil % after PT (*r* = 0.281, *P* value = 0.005), (*r* = 0.339, *P* value < 0.001) respectively (Table 5).

**Table 5:** Correlation between TSB and TNF-α with Eosinophil count and % before and after PT

	**TSB before**		**TNF α before**	
	**r**	***P*-value**	**R**	***P*-value**
TNF α before	0.442	< 0.001*		
Eosinophil count before	-0.114	0.257	-0.055	0.586
Eosinophil % before	0.318	< 0.001*	0.189	0.060
	**TSB after**		**TNF α after**	
	**r**	***P*-value**	**R**	***P*-value**
TNF α after	0.086	0.395		
Eosinophil count after	0.281	0.005*	0.154	0.126
Eosinophil % after	0.339	< 0.001*	0.545	< 0.001*

*TNF α* Tumor Necrosis Factor α, *TSB* Total Serum Bilirubin, *: significant as p-value ≤ 0.05

## Discussion

The most often utilized technique for treating and preventing severe hyperbilirubinemia is PT [14]. Ultraviolet (UV) light exposure begins a complicated cascade of events that results in the immune system being downregulated. Numerous immune mediators such as IL-1, IL-6, IL-10, and TNF-α are released by the immune system of the skin to support the systemic immunologic response [14].

Our results showed a statistically significant decrease in hemoglobin (Hb) after PT. This agrees with Saber et al. [14], where Hb levels were significantly lowered after PT. Also, in Beken et al. [15] study, Hb counts were lower after PT. Furthermore, our results were in line with Can et al. [16], who demonstrated that the total serum bilirubin, hemoglobin, hematocrit, leukocyte, and neutrophil counts were significantly lowered.

While, in El Mashad GM et al. [17] study, there was an insignificant difference between the cases according to hemoglobin level before and after PT.

We also found an insignificant difference in the total leucocytic count before and after PT. This comes in agreement with Saber et al. [14], where a comparison of WBCs count in patients before and after PT showed a lack to show any difference. Also, Kurt et al. [18] stated that WBCs did not reveal any essential changes.

Against our study, Jahanshahifard et al. [19] showed that PT in term neonates could raise peripheral WBC count. Also, in Abdelhakeem et al. [20] study, they observed a significant increase in WBCs after 36 h and after 72 h, then started to decrease after stop of PT on the 7th day. As shown by Can et al. [16] non-significant change in lymphocyte and basophil counts was observed after PT in our study too.

In our finding, there was a decrease in platelet count after PT than before PT. This agrees with Sarkar et al. [21], who demonstrated that platelet count was significantly lower after PT than before. In contrast, Abdel mohsen et al. [22] demonstrated that platelet count was significantly higher after PT than before PT. In our finding, there was a decrease in platelet count after PT than before PT.

We found a significant increase in the absolute and relative eosinophil count after PT. Our finding was in line with the eosinophil count was significantly elevated (*p* = 0.01) after PT. In Beken et al. [15] and El Mashad GM et al. [17] eosinophil levels were also increased after PT for 48–72 h. Altuntas et al. [23] also found that PT was linked with a significant increase in eosinophil.

In the current study, there was a significant increase in tumor necrosis factor α (TNF-α) after PT. This result is in line with Saber et al. [14], who found serum TNF-α levels significantly elevated after exposure to PT and this means the strong effect of PT on TNF-α serum levels.

Our finding agreed with Neam et al. [1], who found that serum TNF-α levels significantly increased after exposure to PT for 72 h when compared to values before PT, demonstrating the influence of PT on serum levels of TNF-α.

Also, Jahanshahifard et al. [19] stated that exposure to PT in the treatment of neonates with hyperbilirubinemia might influence cytokine production and release from the peripheral immune system, as it increases serum TNF-α. Kurt et al. [18] stated that usage of PT in neonates with jaundice as a treatment affects the function of the immune system in newborns through alterations in TNF-α production. Narbutt et al. [24] stated that exposure of healthy term neonates to repeated doses of UV radiations shows a significant increase in serum level of TNF-α. Serum TNF-α and eosinophil count increased after treatment of neonatal hyperbilirubinemia by PT which indicates an allergic response to PT in neonates.

In our finding, there was highly significant positive correlation between TSB and eosinophil % before PT. In agreement with our results, Can et al. [16] found that statistically significant positive correlation between bilirubin and eosinophil levels before PT. Further studies are needed to investigate the relationship between PT and childhood eczema, rhinitis, and early-onset wheezing or allergic sensitization.

### Limitation of the study

The study was a single center with insufficient sample size, and the study did not include the long-term complication of the PT on neonates with hyperbilirubinemia.

So, we recommended that further studies on large scale to evaluate the allergic response of PT also doing the same work in preterm infants for generalization of the results.

Infants receiving PT should be followed up with care to prevent the development of side effects of PT as allergic reactions.

## Conclusions

There was a positive effect of PT on the neonatal serum bilirubin level. This therapeutic modality increased serum TNF- alfa levels that can affect the function of the immune system in newborns. There was a significant positive correlation between total serum bilirubin and both tumor necrosis factor alfa and eosinophil % and between total serum bilirubin and both eosinophil count and eosinophil % after PT.

## Data Availability

Available on reasonable request from the corresponding author.

## Abbreviations

PT: Phototherapy
TNF-α: Tumor necrosis factor α
NICU: Neonatal Intensive Care Unit
Hb: Hemoglobin
TSB: Total serum bilirubin
